# A brain cytokine-independent switch in cortical activity marks the onset of sickness behavior triggered by acute peripheral inflammation

**DOI:** 10.1186/s12974-023-02851-5

**Published:** 2023-07-28

**Authors:** Samu N. Kurki, Tommi Ala-Kurikka, Arto Lipponen, Alexey S. Pospelov, Taisia Rolova, Jari Koistinaho, Juha Voipio, Kai Kaila

**Affiliations:** 1 Faculty of Biological and Environmental Sciences, Molecular and Integrative Biosciences, University of Helsinki, P. O. Box 64, 00014 Helsinki, Finland; 2 Neuroscience Center, HiLIFE, University of Helsinki, Helsinki, Finland; 3 Department of Psychology, University of Jyväskylä, Jyväskylä, Finland

## Abstract

Systemic inflammation triggers protective as well as pro-inflammatory responses in the brain based on neuronal and/or cytokine signaling, and it associates with acutely and protractedly disrupted cognition. However, the multiple mechanisms underlying the peripheral–central inflammatory signaling are still not fully characterized. We used intraperitoneal (i.p.) injection of lipopolysaccharide (LPS) in freely moving mice with chronically implanted electrodes for recording of local field potentials (LFP) and electrocorticography (ECoG) in the hippocampus and neocortex, respectively. We show here that a sudden switch in the mode of network activity occurred in both areas starting at 10–15 min after the LPS injection, simultaneously with a robust change from exploration to sickness behavior. This switch in cortical mode commenced before any elevations in pro-inflammatory cytokines IL-1β, TNFα, CCL2 or IL-6 were detected in brain tissue. Thereafter, this mode dominated cortical activity for the recording period of 3 h, except for a partial and transient recovery around 40 min post-LPS. These effects were closely paralleled by changes in ECoG spectral entropy. Continuous recordings for up to 72 h showed a protracted attenuation in hippocampal activity, while neocortical activity recovered after 48 h. The acute sickness behavior recovered by 72 h post-LPS. Notably, urethane (1.3 mg/kg) administered prior to LPS blocked the early effect of LPS on cortical activity. However, experiments under urethane anesthesia which were started 24 h post-LPS (with neuroinflammation fully developed before application of urethane) showed that both theta–supratheta and fast gamma CA1 activity were reduced, DG delta activity was increased, and sharp-wave ripples were abolished. Finally, we observed that experimental compensation of inflammation-induced hypothermia 24–48 h post-LPS promoted seizures and status epilepticus; and that LPS decreased the threshold of kainate-provoked seizures beyond the duration of acute sickness behavior indicating post-acute inflammatory hyperexcitability. Taken together, the strikingly fast development and initial independence of brain cytokines of the LPS-induced cortical mode, its spectral characteristics and simultaneity in hippocampus and neocortex, as well as inhibition by pre-applied urethane, strongly suggest that the underlying mechanisms are based on activation of the afferent vagus nerve and its mainly cholinergic ascending projections to higher brain areas.

**Supplementary Information:**

The online version contains supplementary material available at 10.1186/s12974-023-02851-5.

## Background

Animals respond to threatening pathogens by activation of an innate immune response, inflammation, which activates immune cells as well as peripheral and central neuronal mechanisms to adapt behavior for enhanced probability of survival [1–3]. An identical response can be induced by sterile inflammation (i.e., with no pathogen present), underscoring the stimulus-independence and convergence of mechanistic cascades activated across all triggers of innate immunity (e.g., infection, physical trauma, or ischemic event) [4, 5]. However, this potent response which often leads to a hyperinflammatory cytokine storm and sepsis, can be life-threatening to the host itself [6, 7]. Beyond acute mortality, cytokine storm and sepsis are associated with severe long-term sequelae leading to poor functional outcome [8–10].

Acutely disrupted cognitive function and delirium are brain-specific manifestations of systemic inflammation that promotes neuroinflammation [8, 11, 12]. Patients surviving critical illness often suffer significant cognitive impairment such as compromised memory and executive function, as well as extensive and protracted fatigue [9, 13]. Recent studies have revealed similar disabilities in patients recovering from the COVID-19 disease [14]. A rapidly increasing amount of evidence points to a tight link between neuroinflammation and the development of chronic neurodegenerative conditions such as Alzheimer’s disease [15–17]. Thus, there is an urgent need for more information on the mechanisms triggering and mediating the deleterious central effects of systemic inflammation, for which there are currently no approved therapies [18].

The brain receives neuroinflammatory signals mediated by cytokines via the circulatory system [19, 20], and from neurons in the brainstem and hypothalamus which are activated promptly by cytokines after a peripheral inflammatory stimulus [21–23]. In addition, the vagus nerve is an important and direct route for afferent immune-to-brain signaling [24]. Several studies have been done in order to identify those cytokines which might act as the initial sensors within the brain in response to an inflammatory threat. For instance, Duan et al. [20] posit that chemokine CCL2 secreted by PDGFRb-positive mural cells of brain vasculature is the first inflammatory signal relayed to the brain after a peripheral trigger with a delay of 2 h. However, the more recent work by Osterhout et al. [21] and Ilanges et al. [22] contradicts with this view by showing (based on increased *Fos* expression) that multiple deep brain areas controlling basic physiology such as body temperature and thirst are affected within 1 h after the inflammatory trigger.

In the present study, we aimed to find out the delay from the induction of peripheral inflammation to subsequent effects on the brain. In particular, we were interested in effects on cortical structures because they are not only targets of pathophysiological inflammatory cascades, but they also control ‘higher-order’ predictive/adaptive functions at early stages of inflammation [25]. We examined changes in the spontaneous activity by recording local field potentials (LFP) and electrocorticography (ECoG) signals in the hippocampus (HC) and neocortex (NCX) of freely moving mice immediately following lipopolysaccharide (LPS) injection intraperitoneally (i.p.), which models sterile inflammation and was used in all of the four studies described above [19–22]. In parallel, we measured cytokine levels in blood and hippocampal samples at several time points. We show here that the latency from LPS injection to a robust switch in the mode of hippocampal and neocortical activity was only about 10 min. This delay is significantly shorter than what has been previously reported and, notably, the hippocampal and neocortical responses preceded the elevations of pro-inflammatory cytokines in the brain parenchyma.

In view of the novelty of the above observations, we were interested in the temporal progression of the central effects during the full time course (presently ~ 72 h) of the onset and recovery of sickness behavior [26] and beyond. A previous study with peripheral LPS injection found a decreased frequency of hippocampal CA1 theta oscillations during the first 24 h post-injection (hpi), paralleled by enhanced delta activity [27]. We substantially extended these findings and demonstrate in experiments on anesthetized animals that the inflammation-induced disturbance in hippocampal function significantly suppresses sharp-wave ripples (SPW-R) in CA1, but evokes enhanced slow-wave activity in the dentate gyrus (DG). Finally, we found that experimental compensation of LPS-induced hypothermia promoted seizures and status epilepticus, and that there was a marked decrease in seizure threshold after the recovery of acute sickness behavior, pointing to hyperexcitability as a chronic sequela during and after acute systemic inflammation [28–30].

## Results

### Intraperitoneal LPS injection affects spontaneous cortical activity with a surprisingly early and abrupt onset

**Fig. 1:**
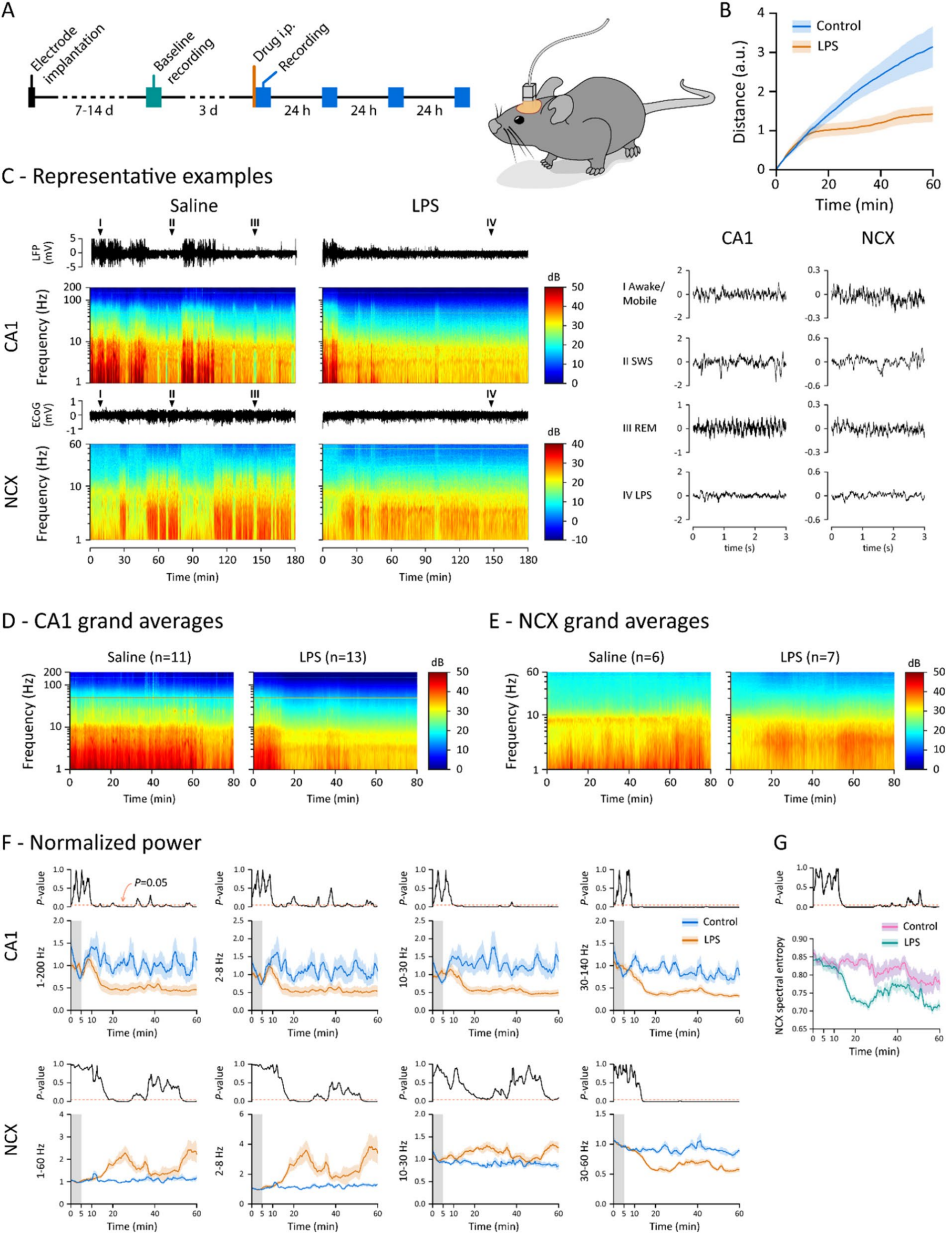
Peripheral inflammatory stimulus induces a sudden switch in hippocampal and neocortical activity paralleled by a change in behavioral mode. **A** Schematic of the experiment. Mice with chronically implanted electrodes were given saline or LPS i.p. and their spontaneous brain activity and behavior were recorded with a video-LFP/ECoG system starting immediately after the injection for the following 3 h, and continuing thereafter at 24, 48 and 72 h post-injection as shown in Fig. 3. n is 11–15 except for 72 h where n is 5. **B** Cumulative sum of traveled distance during the first hour after saline (n = 11) or LPS (n = 13) injection. **C** Representative data following saline or LPS injection: raw LFP/ECoG traces and corresponding spectrograms (time–frequency plots) across the 3-h follow-up period. Arrowheads point to timepoints during cortical modes I–IV with corresponding 3 s raw traces shown on the right. **D**, **E** Grand average of spectrograms of the whole experimental cohort receiving saline (**D**) or LPS (**E**) injection (*n* = 11 and 13, respectively). **F** Time evolution of CA1 LFP and NCX ECoG power spectra during the first hpi within the frequency range of 1–200 Hz and separated into three different frequency bands as indicated. Power was normalized to the first 5 min after injection. **G** Time evolution of ECoG spectral entropy during the first hpi. Continuous black traces show p values over time from *t*-tests

To assess the earliest responses in the cortex to a developing systemic inflammation, we first studied the LFP activity in the HC CA1 subfield and ECoG in the frontal somatosensory cortex in freely moving mice (Fig. 1). Both the CA1 and NCX display three different oscillatory modes under baseline conditions, corresponding to the behavioral states of the animal confirmed on the basis of video recordings: awake/mobile (Fig. 1C, mode I), slow-wave sleep (SWS; mode II), and short bouts of rapid eye movement (REM) sleep amidst the SWS (mode III). These well-known modes have readily discernible power spectra, with characteristic peaks at theta frequency band (4–12 Hz) in modes I and III (Additional file 1: Fig. S1).

We used a protocol in which the animal is injected i.p. with saline or 2 mg/kg LPS just before introducing it to the recording cage with fresh bedding. This immediately and reliably evoked a lengthy period of exploratory behavior with a stable cortical mode I, thus permitting an unambiguous identification of the effects of LPS on CA1 and NCX. Saline-injected animals (n = 11) kept exploring the cage for approximately 60 min before digging a nest-like pit into the bedding and falling asleep, as seen by full immobility and mode II (Fig. 1B, C). In stark contrast to this, the LPS-injected animals (*n* = 13) abruptly halted their exploration and froze with a latency of 10–15 min (Fig. 1B). A more accurate and unambiguous estimate of the onset of the central effect was determined on the basis of the electrophysiological recordings.

**Fig. 2:**
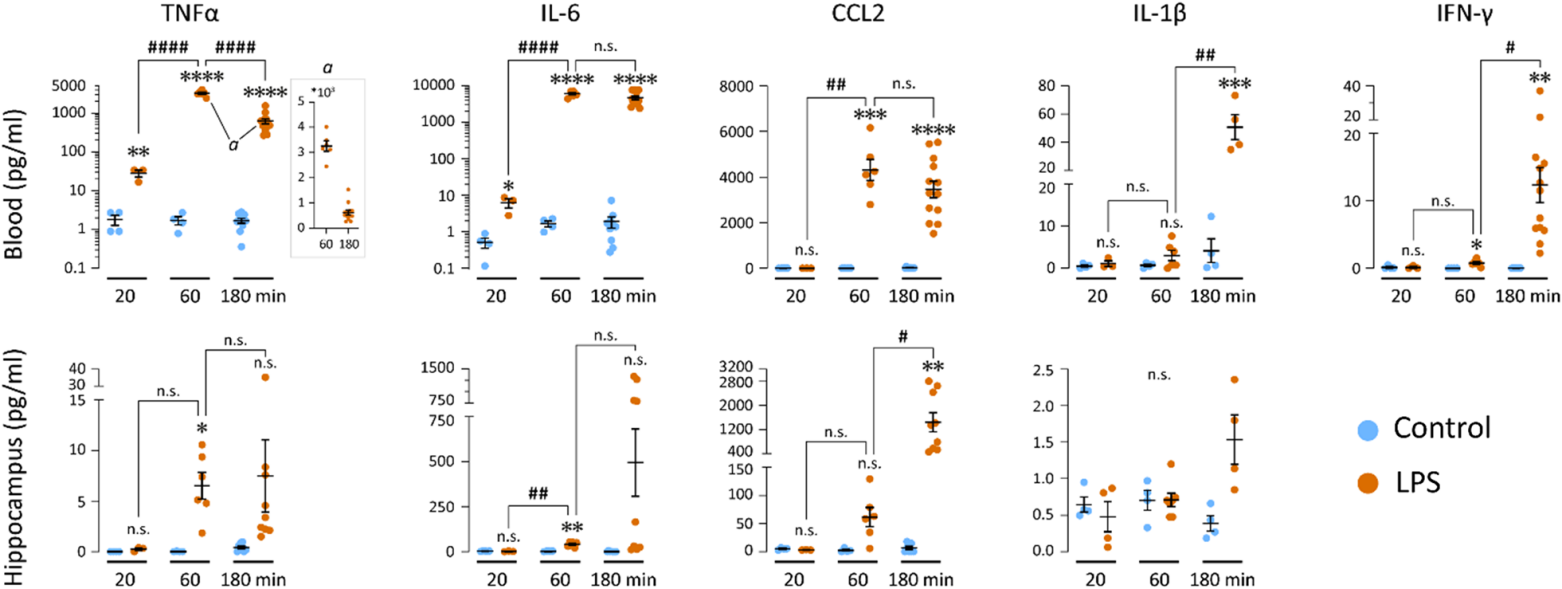
Time course of cytokine responses in blood plasma and hippocampal tissue following LPS injection. Concentration of TNFα, IL-6, CCL2, IL-1β and IFN-γ 20 min, 1 h and 3 h after saline (n is 4–10) or LPS (n is 3–13) injection in blood plasma and hippocampus. Note the absence of brain cytokine elevation at 20 min post-LPS. Statistical testing by unpaired t-tests with Holm–Sidak’s correction for multiple comparisons. * and # denote comparisons between saline and LPS groups at the same timepoint and between LPS groups, respectively; ****^/####^*p* < 0.0001, ***^/###^*p* < 0.001, **^/##^*p* < 0.01, *^/#^*p* < 0.05, n.s. not significant

Simultaneously with the behavioral change, a sudden switch in cortical activity occurred, which was first visible as significantly attenuated CA1 LFP gamma (30–140 Hz) power and a simultaneous fall of the neocortical ECoG gamma (30–60 Hz) power starting at no later than 10 min after the LPS injection (Fig. 1). At 11 min after the injection, the decrease in CA1 power was significant across the whole frequency spectrum (1–200 Hz). In the NCX the reduction of gamma power (30–60 Hz) was associated with a significant increase at low frequencies (2–8 Hz). Notably, these experiments show that the delay from the intraperitoneal LPS injection to the consequent alterations in cortical activity (about 10 min) is much shorter than those of the previously reported early brain responses to systemic inflammation (see “Background” section). Most importantly, our cytokine assays showed that *the LPS-triggered effects on cortical activity start before any pro-inflammatory cytokines are elevated in the brain parenchyma* (Fig. 2).

The cortical state provoked by LPS (mode IV) was different from both the active and inactive modes (Fig. 1C and Additional file 1: Fig. S1, mode IV): both the hippocampal LFP and neocortical ECoG showed broadband attenuation compared to the SWS mode II in controls, which was selected as a reference because the animals were in a fully immobile state after LPS. Both brain areas also displayed enhanced oscillations at approximately 3 Hz, which were probably coupled to breathing [31, 32]. There was a brief period around 40 min after the injection when the animals showed a transient partial recovery with locomotion and enhanced hippocampal and neocortical activity (Fig. 1F). Otherwise, the inflammatory state (mode IV) was dominant over the first 3 h after LPS injection in 12 out of 13 animals.

Spectral power is a robust parameter of neuronal population activity, and therefore well-suited for the aims of the present work. We also analyzed changes in spectral entropy (SpE; see “Discussion” section), which is a non-linear measure of signal irregularity independently of the signal amplitude [33]. Notably, LPS induced a prominent decrease in neocortical ECoG SpE. The transient recovery of behavior at around 40 min after the injection was associated with a parallel increase of SpE (Fig. 1G).

### Time course of cytokine responses in blood and brain tissue following LPS injection

Next, we carried out cytokine assays in blood and brain (hippocampal) tissue to examine whether the sudden brain response to peripheral LPS might be caused by the previously identified earliest mediators of innate immune activation [19–22], including the pro-inflammatory cytokines interleukin-1beta (IL-1β), tumor necrosis factor alpha (TNFα), C–C motif chemokine ligand 2 (CCL2) and interleukin-6 (IL-6) (see “Background” section). In addition, we measured levels of interferon-gamma (IFN-γ), a key early mediator of antigen presentation and leukocyte trafficking [34].

Strikingly, at 20 min post-injection, the timepoint when the fast-onset cortical response was fully established in all animals tested, none of the above cytokines was elevated in hippocampus (Fig. 2). In blood plasma, TNFα and IL-6 were slightly elevated by this time, albeit still to low levels, indicating the onset of activation of the systemic immune response (Fig. 2). Notably, CCL2, which has been suggested to first relay the inflammatory signal from the PDGFRb-positive cells to the central nervous system [20], showed no increase in blood plasma by this time, and neither did IL-1β nor IFN-γ.

At 1 hpi, there was surge in both the plasma and hippocampal concentrations of TNFα, CCL2 and IL-6. IL-1β was not significantly increased in the plasma nor the hippocampus. IFN-γ was slightly but significantly elevated in the blood, but undetectable in the hippocampus (Fig. 2).

At 3 hpi, blood CCL2 and IL-6 reached very high levels, while TNFα had already significantly plummeted from its maximum at 1 hpi. IL-1β and IFN-γ also showed a clear elevation at this timepoint (Fig. 2). In the brain, CCL2 and IL-6 concentrations continued to rise strongly, TNFα maintained its elevated level, and IL-1β was also noticeably increased compared to saline (Fig. 2).

### LPS induces a prolonged attenuation of hippocampal and cortical brain activity

**Fig. 3:**
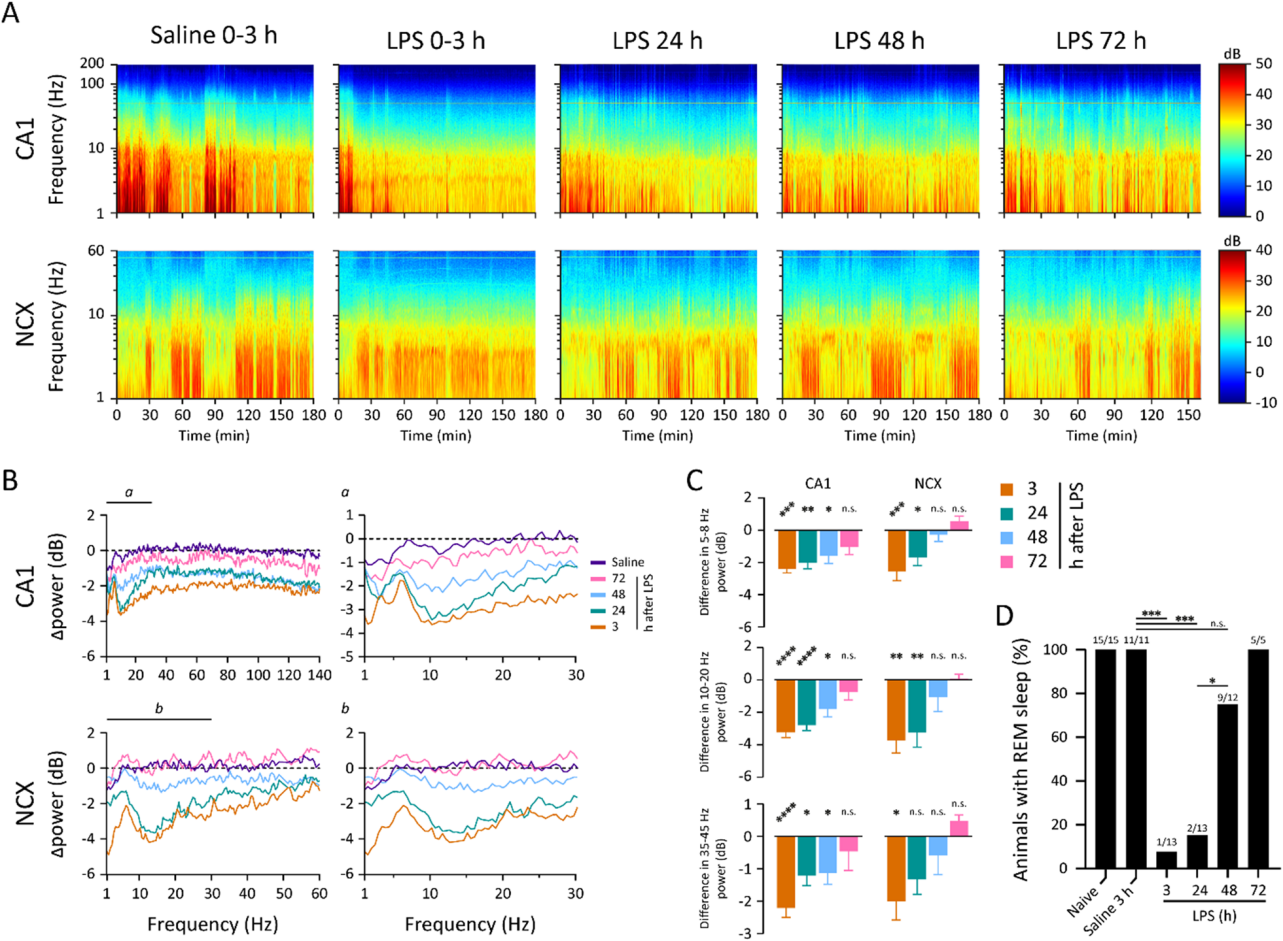
Prolonged effects on cortical activity induced by neuroinflammation in freely moving animals. **A** Representative spectrograms from a single animal during five consecutive recording periods from saline injection to 72 h post-LPS. **B**, **C** CA1 and NCX power spectra (**B**) and quantitative bar graphs taken thereof in various frequency bands (**C**) display a specific and prolonged attenuation pattern. Statistical testing by one-way ANOVA followed by Dunnett post hoc tests. **D** REM sleep is abolished early after LPS and recovers protractedly. Statistical testing by Fisher’s exact test. ****p < 0.0001, ***p < 0.001, **p < 0.01, *p < 0.05, n.s. not significant. For n numbers see Fig. 1

**Fig. 4:**
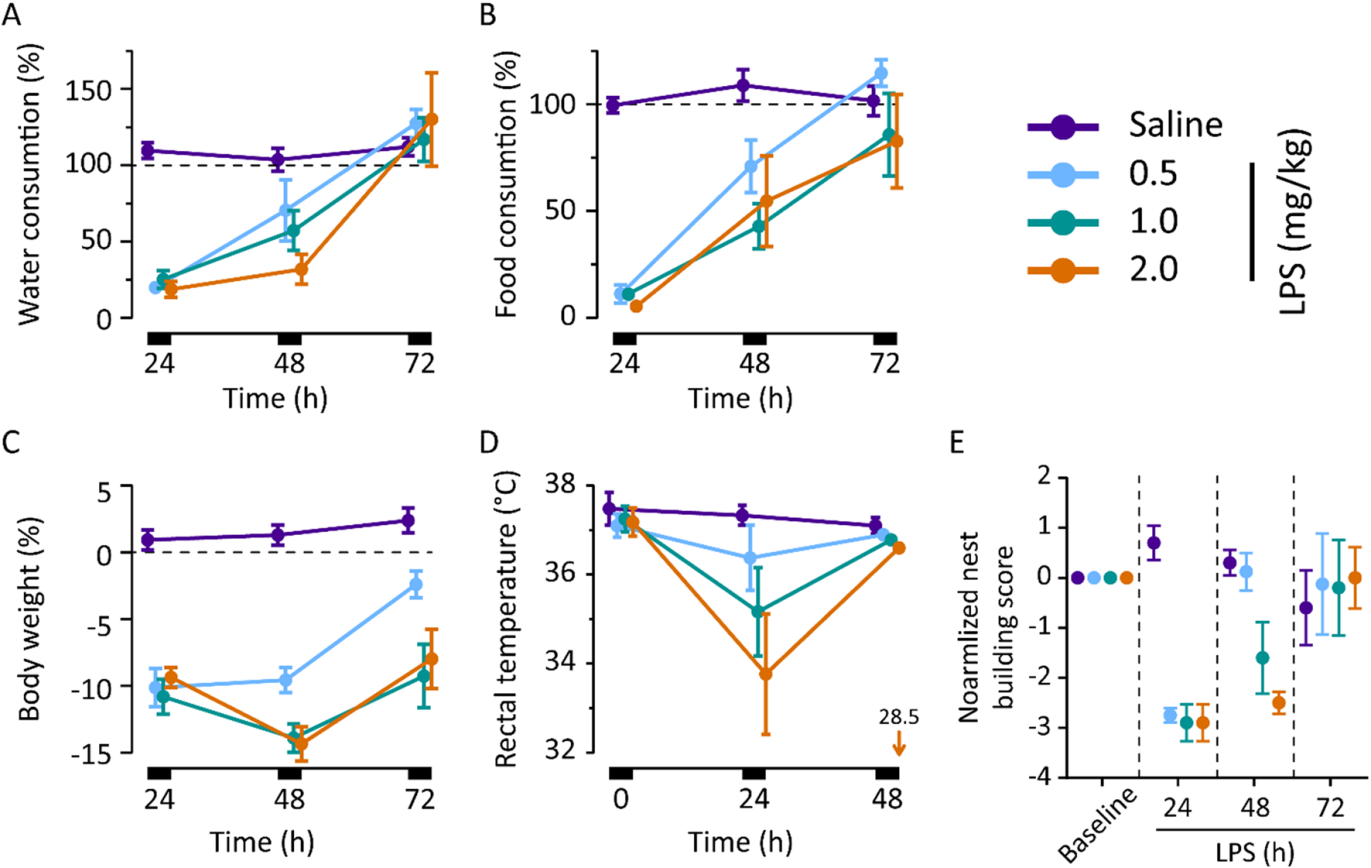
Peripherally applied LPS induces sickness behavior which dose-dependently resolves by 72 h. **A**–**E** Indicators of sickness and sickness behavior in LPS-injected animals (0.5, 1.0 and 2.0 mg/kg LPS): **A** water consumption, **B** food consumption, **C** body weight, **D** rectal temperature and **E** nest building. Statistical test values and n numbers are provided in Additional file 2: Table S1

Next, we examined the more prolonged effects of LPS on cortical activity (Fig. 3), as well as the onset and progression of sickness behavior (Fig. 4) for 72 hpi using the same freely moving mice with chronically implanted electrodes as in Fig. 1. At 3 hpi, the CA1-LFP and NCX-ECoG power were attenuated in LPS-injected animals across all frequency bands, with the strongest effect at slow delta (1–2 Hz) and supratheta (10–20 Hz) frequencies. Notably, the attenuation was considerably smaller in the central theta band (5–8 Hz), and to a lesser extent at 3–4 Hz band (Fig. 3B, C). Gamma frequency activity was attenuated across the entire band (CA1 30–140 Hz; NCX 30–60 Hz, higher frequencies were not reliably collected due to the large surface of the NCX electrode).

After 24 hpi, the power of slow delta in the LPS group had recovered relatively more than theta and supratheta, which had a highly similar relative attenuation as seen at 3 hpi. Gamma activity was recovering at a similar rate in both CA1 and NCX (Fig. 3B, C).

By 48 h after LPS, the relatively smaller (compared to slow delta and supratheta) although significant attenuation of theta activity compared to nearby frequencies was still detectable in CA1, but not in NCX power spectra. The supratheta and gamma power in CA1 did not recover after 24 hpi unlike in the NCX (Fig. 3B, C).

At the last follow-up time window, starting at 72 h after LPS, there was no significant attenuation in either brain area in any frequency band (Fig. 3B, C).

REM sleep was promptly and transiently abolished during the first 24 h post-LPS (Fig. 3D), which confirms and extends previously reported findings ([35] and references therein).

### LPS-induced sickness behavior recovers by 72 h after LPS

While peripheral LPS induces sickness behavior in a reliable and dose-dependent manner [36], its efficacy is known to vary a lot depending on the source of LPS and strain of mice [37, 38]. Thus, we carried out behavioral and physiological experiments to get an idea of the severity of the inflammation induced by the 2 mg/kg of LPS used presently. Specifically, we measured motivated behaviors including drinking, eating and nest building, as well as body weight and rectal temperature. To further calibrate the effect of our default LPS dose of 2 mg/kg with relation to previous studies ([19], e.g., [20, 39]), we also tested 0.5 and 1 mg/kg of LPS in these experiments (Fig. 4, Additional file 2: Table S1).

We found that all doses of LPS examined (0.5, 1 and 2 mg/kg i.p.; *n* = 4, 6 and 6, respectively) induced an almost total cessation of drinking and eating during the first 24 hpi, and recovery was observed in all groups by 48 hpi. Full recovery of drinking and eating took place within about 72 hpi (Fig. 4A, B). The weight of the animals fell by ~ 10% by 24 hpi in all groups, while the recovery showed dose-dependently diverging tracks by 48 hpi, with the LPS 0.5 mg/kg group starting to gain weight, while the higher dose groups were still losing it (Fig. 4C). By 72 hpi, all groups had started recovering weight. The mean rectal temperature of the animals fell by 24 hpi with a consistent but non-significant dose-dependent trend (Fig. 4D), and had recovered to normothermia (> 36.0 °C) except for one animal (in the LPS 2 mg/kg group) by 48 hpi. None of the animals had fever (> 38.5 °C) at any time point. Nest building behavior was absent at 24 hpi in all LPS-injected animals, regardless of dose. A dose-dependent recovery commenced at 48 hpi and was complete in all groups by 72 hpi (Fig. 4E). Together, these results demonstrate that with a temporal resolution of 24 h, LPS-injected animals have a functional nadir at around 24 h post-LPS, and have essentially recovered from acute sickness by 72 hpi.

### Urethane blocks the acute effects of LPS on hippocampal activity

**Fig. 5:**
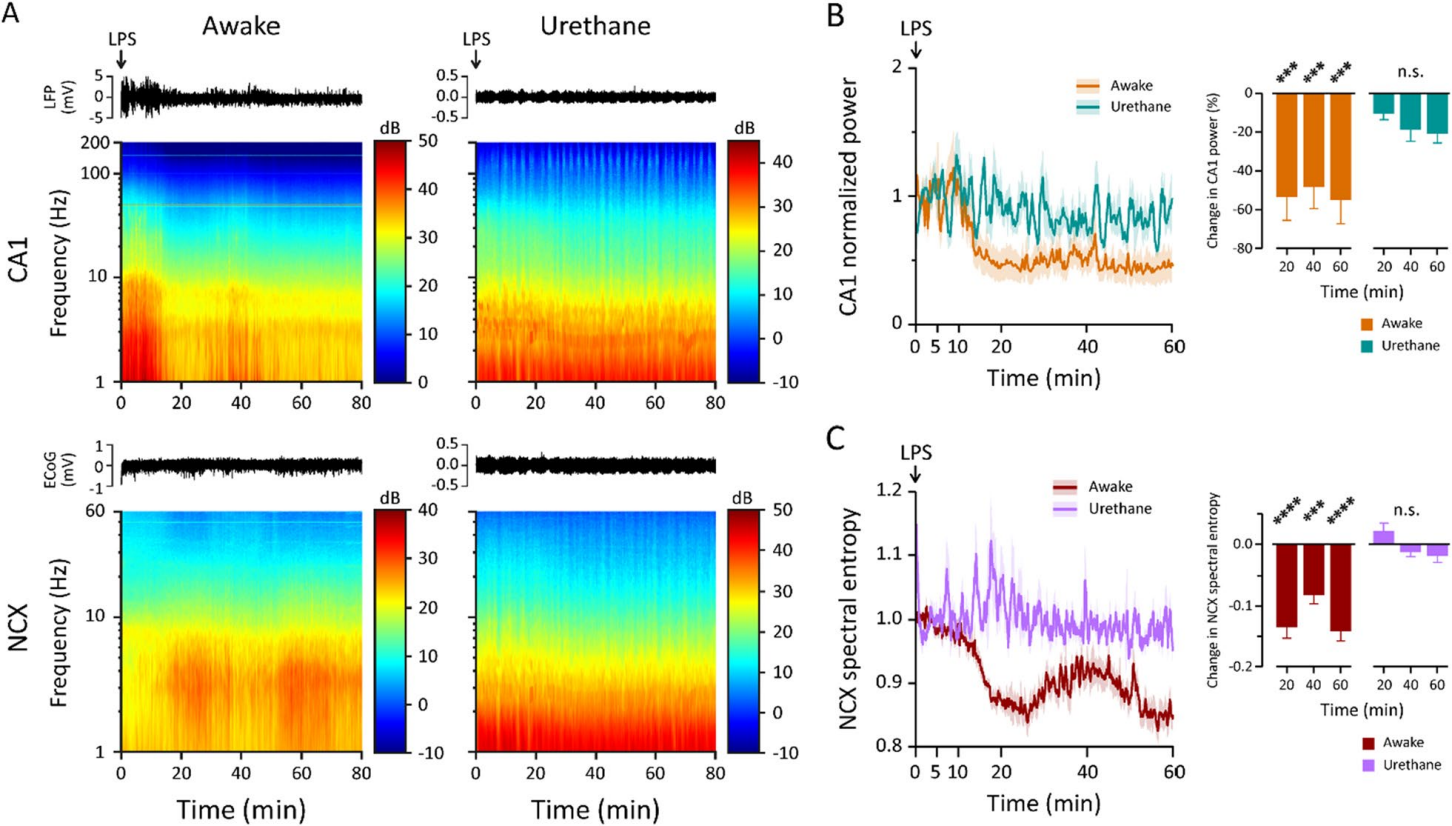
Urethane-anesthesia blocks the fast action of LPS on cortical state. **A** Representative raw traces and grand average spectrograms of CA1 and NCX response to LPS injected at time zero in awake and urethane-anesthetized animals (n is 13 and 5 in CA1; 7 and 4 in NCX, respectively). **B** Normalized power at 1–200 Hz in CA1 was significantly reduced after LPS injection in awake animals, but not in animals under urethane anesthesia. **C** Normalized NCX SpE was significantly reduced after LPS injection in awake animals, but not in animals under urethane anesthesia. Statistical testing performed with two-way ANOVA followed by Dunnett post hoc tests; ****p < 0.0001, ***p < 0.001, n.s. not significant compared to the first 5 min period after LPS injection

As another approach to look at the acute central effects of LPS, we studied animals under urethane anesthesia. Strikingly, and unexpectedly, urethane (1.3 mg/kg i.p.) anesthesia preceding the LPS injection *completely blocked* the sudden-onset change in cortical state seen in the awake animals as is evident from the spectrograms (Fig. 5A). This lack of effect is clearly seen in CA1 power (Fig. 5B). Because the NCX power in the low and high-frequency bands responded to LPS in opposite manners (unlike the CA1 power, see Fig. 1D–F), we used SpE as a further indicator of the NCX LPS response, which also showed a block of LPS effect (Fig. 5C). No effect of the LPS injection could be detected in either CA1 or NCX during the one-hour recording period in these experiments. The absence of LPS effects is likely due to the actions of urethane on the cholinergic pathways ([40]; see “Discussion” section).

### 24 h post-LPS, both theta–supratheta and fast gamma CA1 activity are reduced, DG delta activity is increased, and SPW-R are abolished in experiments under acute urethane anesthesia

**Fig. 6:**
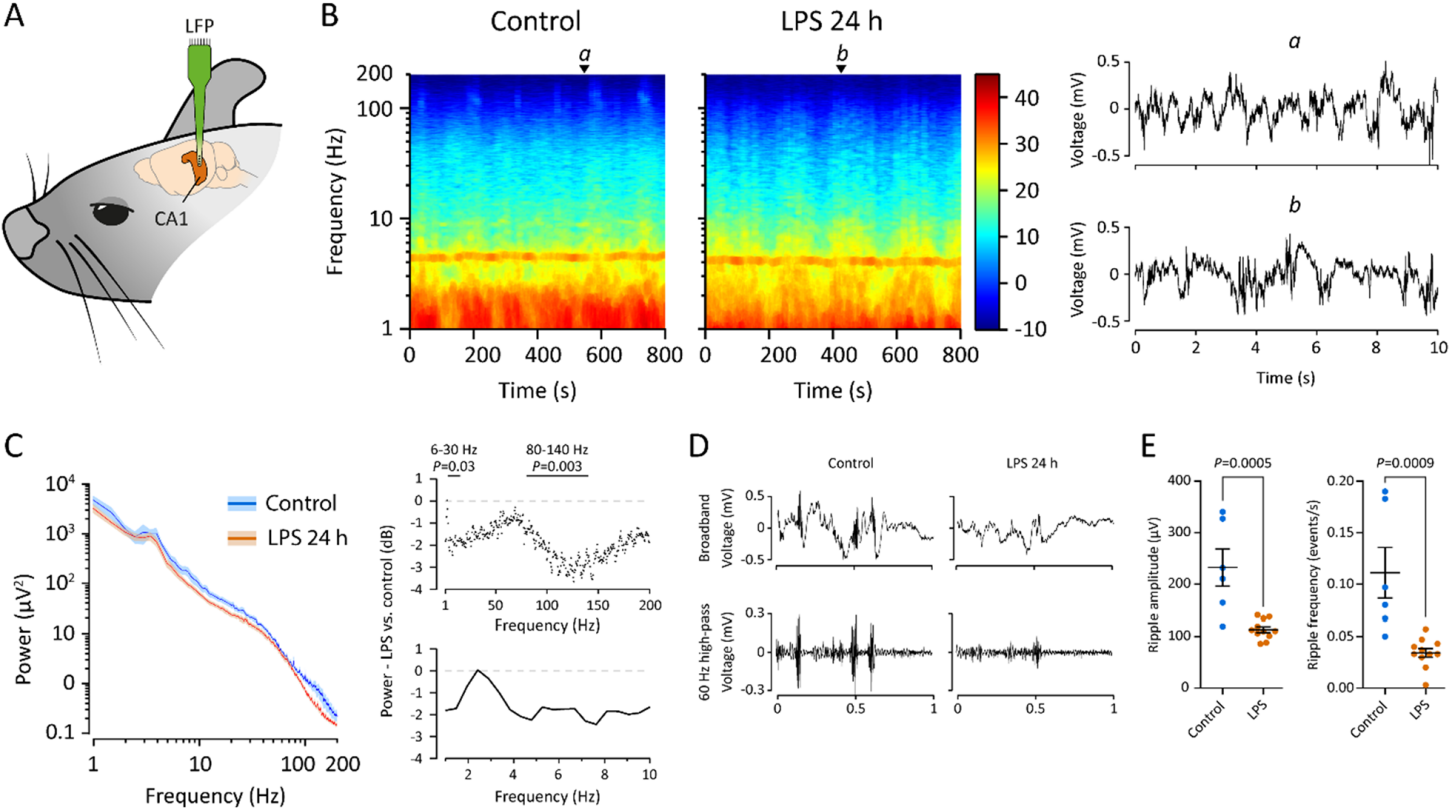
Under urethane anesthesia starting 24 h post-LPS, the fully developed neuroinflammation attenuates spontaneous CA1 activity and abolishes SPW-R. **A** Site of recording. **B** Spectrogram and raw traces of spontaneous CA1 activity under urethane anesthesia 24 h after saline or LPS injection. **C** Power spectra of the CA1 activity 24 hpi demonstrated significant attenuation in the theta–supratheta (6–30 Hz) and fast gamma (80–140 Hz) frequency bands; statistical testing by unpaired *t*-test. **D** Representative SPW-R in raw and filtered traces after saline or LPS injection. **E** SPW-Rs were strongly suppressed after LPS both in amplitude and frequency; statistical testing by t-test. n is 6 and 11 in saline and LPS groups, respectively

We next examined more closely the protracted effects of LPS injection on hippocampal activity and excitability. These experiments were done under urethane anesthesia using multitrode electrodes for simultaneous recording in CA1 and dentate gyrus (DG) (Fig. 6A, B, Additional file 1: Fig. S2A) starting at 24 hpi, i.e., the timepoint when the LPS-induced sickness behavior was fully developed in non-instrumented animals (see Fig. 4). It is important to point out here that the suppressing effects of urethane on LPS-induced inflammation disappear when urethane is given > 2 h after the LPS [41]. Moreover, doing these experiments under anesthesia allowed us to stabilize the core temperature (cf. Fig. 4D) of the animals during the recordings. In contrast to the experiments in the previous section in which the mice were anesthetized prior to the LPS injection, the animals were now first injected with LPS or saline, and 24 h later anesthetized with urethane for the multitrode experiments (Fig. 6A).

**Fig. 7:**
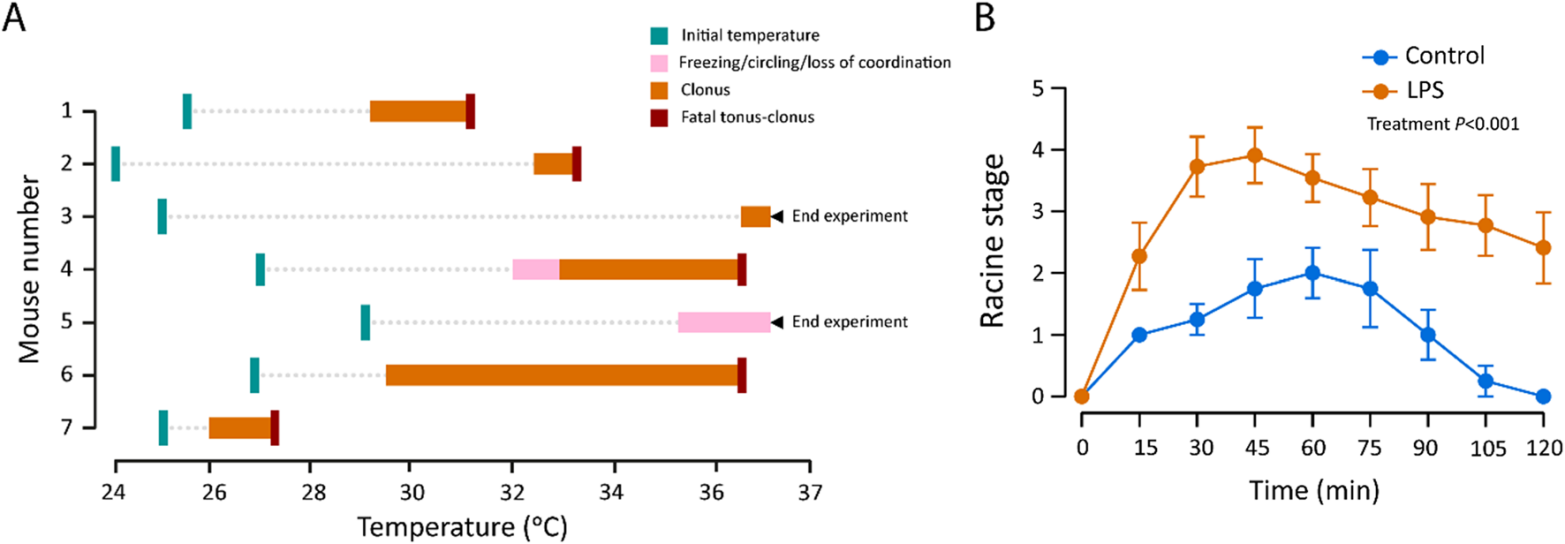
LPS-triggered neuroinflammation significantly decreases threshold to kainic acid-provoked seizures 72 h after injection, when the behavioral sickness has already resolved. **A** Slow gradual rewarming (over a period of 20–30 min) of mice during LPS-induced hypothermia (see Fig. 4) to a maximum body temperature of 36.5 °C triggered seizures at levels of body temperature below normothermia. For details see main text. **B** Kainate-induced seizures as quantified using the Racine scale were more severe 72 h post-LPS vs controls; n is 4 and 11 in saline and LPS groups, respectively. Statistical testing by two-way ANOVA

We found a moderate broadband suppression of CA1 LFP (Fig. 6C), which was most prominent in the theta–supratheta band (6–30 Hz) and high gamma band (80–140 Hz). In view of the seizure-promoting actions of LPS treatment (see below Fig. 7), it was interesting to note that in the DG, the LFP activity was increased in the delta frequency band (1–4 Hz; Additional file 1: Fig. S2A), unlike what was observed in CA1.

Strikingly, we also found that the SPW-R, which specifically appear in CA1 and are considered as key network events in memory encoding and retrieval [42], were strongly suppressed at 24 h after LPS (Fig. 6E). Finally, we analyzed a subset of LFP multitrode recordings from the deep layers of parietal cortex. Here, we observed suppression of cortical up–down state transitions [43, 44], which was directly visible in the raw traces and as an almost order-of-magnitude attenuation within the 1–4 Hz frequency band. In line with the suppression of up–down state transitions, the fast gamma (80–140 Hz) activity was also significantly attenuated (Additional file 1: Fig. S2B).

### Acute neuroinflammation promotes status epilepticus and decreases the threshold of kainate-provoked seizures beyond the duration of acute sickness behavior

Brain inflammation is considered a major contributor to epileptogenesis [45]. In our experiments with chronically implanted electrodes, two mice had fatal status epilepticus (SE) when they were gently warmed up from deep hypothermia (around 25–28 °C) on a 37 °C water pad (Fig. 7A, animals #1 and #2). Notably, both SE events took place at a level of physiological hypothermia of < 33 °C, which strongly suppresses the excitability of a healthy brain [46]. We tested the effect of a gradual warm-up in 5 non-instrumented mice which had developed a hypothermia below 30 °C in response to LPS at 24 hpi; of these, three had fatal SE at core temperatures no higher than 36.5 °C (Fig. 7A, animals #3–#7). To our best knowledge, these are the first data on systemic LPS application leading to seizures in the absence of established proconvulsant conditions (cf. [47, 48]).

We further examined the behavioral seizure proneness induced by LPS with non-instrumented mice using kainate (15 mg/kg i.p.) at 72 h after saline or LPS. This is a time point when there is little or no overt sickness behavior anymore and the animals were normothermic (see Fig. 4). Seizures in the LPS group were significantly more severe (Fig. 7B). These data provide further evidence that the neuroinflammatory perturbation of cortical excitability caused by a single injection of LPS extends beyond the acute sickness behavior.

## Discussion

Identification of the mechanisms whereby systemic inflammation leads to neuroinflammation [49] is an area of research with exceptionally high translational impact. The main finding of the present work is that intraperitoneally applied LPS causes a surprisingly fast and well-defined switch in hippocampal and neocortical state, with an onset in around 10 min after the injection. This effect was robust and showed little inter-individual variation. Notably, these central effects *preceded* the severe neuroinflammation triggered by LPS, as evidenced by the absence of elevation of cytokine levels in hippocampal tissue at this early time point. Indeed, even with the high sensitivity of the current assays (see “Methods” section), there was no detectable elevation in the HC tissue samples of any of the classically known first-responder cytokines even at 20 min post-injection. To our best knowledge, this kind of a brain cytokine-independent short-latency effect has not been described before. Blood plasma TNFα concentration was already elevated by this time, indicating production from peritoneal macrophages into the ascites fluid, which has been shown to occur within about 15 min following intraperitoneal LPS [50].

The LFP and ECoG recordings in the HC and NCX, respectively, showed three well-known modes of activity under control conditions (modes I–III), corresponding to awake mobility, SWS and REM sleep. While we could detect these modes based on their differing spectral profiles matched with the behavior in synchronized video recording, the addition of an electromyographic (EMG) channel to our video-EEG system would obviously have enabled a more accurate distinction of e.g., awake immobile and SWS states [51], which are difficult to separate based on EEG and behavior alone. The earliest effect of LPS was seen as a robust change in spectral profile of HC LFP and NCX ECoG activity (temporally co-occurring with behavioral freezing), followed by a prompt transition to another (“inflammatory”) cortical state (mode IV). Again, inter-individual variation was negligible, and there was no observable difference in the post-LPS delays in the onset of the transition from state I to IV between the HC and NCX. The invariant temporal properties of the initial “switch” which evolved 10–15 min post-LPS with a transition from I to IV are mediated by the afferent arc of the immune reflex [24], i.e., the vagus nerve, as will be discussed in detail below.

Cortical mode IV was dominant for the rest of the duration of the 180 min recording sessions in the freely moving animals, except for a brief (~ 10 min) period of partial recovery at around 40 min post-injection for which our data do not provide an explanation. In this context, it is important to note that we did not identify any short-latency effects of the profound elevations in individual brain cytokine concentrations on the LFP and ECoG power in the HC and NCX. Mode IV was characterized by a broadband HC and NCX attenuation except a relative enhancement of activity at ~ 3–3.5 Hz (mostly HC) and 6 Hz (both areas) compared to the behaviorally identical (full immobility) mode II.

Interestingly, cortical mode IV was reflected in a highly similar changes (sustained decline starting from 15 min and a short partial recovery centered at 40 min) in cortical SpE. Notably, SpE quantifies the spectral complexity of the ECoG signal independently of signal amplitude, and is therefore an orthogonal measure to the signal power. Spectral entropy of the electroencephalogram (EEG) is routinely used to monitor the level of consciousness of patients under general anesthesia [52], with values lower than baseline signifying deeper sedation, and higher than baseline values connected to an elevated level of consciousness [53]. Thus, the present SpE data indicate a decreased cognitive alertness following LPS. EEG has previously demonstrated usefulness in monitoring patients with systemic inflammation: human sepsis patients showed excessive theta activity in EEG [54] and a vast majority of COVID patients hospitalized and under EEG registration had abnormalities [55]. In the present study, the cortical response to LPS induced a prominent change in spectral entropy. As SpE is readily available in widely used intensive care monitors, our data suggest that it could be useful in follow-up of septic patients, providing novel information on cortical recovery or impending relapse.

Activation of the afferent vagus nerve is the most parsimonious explanation for the early onset and establishment of mode IV for several reasons:

1. The vagus transmits inflammatory signals almost immediately after a peritoneal challenge of TNFα [56]. The delay from intraperitoneal administration of LPS to its central effect in our experiments would be compatible with the release and direct vagal targeting of early pro-inflammatory cytokines such as IL-1β and TNFα from peritoneal macrophages. In addition, the vagus is directly sensitive to LPS itself at the jugular nodose ganglion [57], and could therefore be also activated by LPS absorbed from the peritoneal cavity into the bloodstream.
2. Ilanges and colleagues [22] recently reported early c-Fos upregulation following i.p. LPS in the nucleus of the solitary tract (NTS), which directly receives afferent signals from the vagus, and has output connections to hypothalamus and brainstem nuclei which are equally activated by systemic application of LPS [21, 58]. Notably, the ascending cholinergic pathways from the brainstem nuclei target both the HC and NCX, thereby explaining the simultaneous onset of the initial effects of LPS in these two brain regions [59].
3. Medial septal cholinergic activation with parallel GABAergic signaling can be induced by stimulation of the vagus [60, 61], which leads to a strong suppression of slow delta and supratheta frequencies, paralleled by a relative enhancement of theta power [62]. This is exactly the kind of a spectral signature that was observed in the present study during the first 24 h of systemic inflammation.

In the broad context of the immune–brain signaling the vagus has a well-established role in via the inflammatory reflex whereby local activation of afferent vagal nerves by IL-1β and TNFα leads to the release of acetylcholine (ACh) into the major internal organs (e.g., liver, heart, spleen and gastrointestinal tract) [63–65]. ACh then stimulates α7-subtype nicotinic ACh-receptors on local tissue macrophages, which quenches their release of pro-inflammatory cytokines to protect the host [24]. It is important however, that these ACh-mediated effects are different from the afferent vagal signaling to the brain which is based on numerous additional neurotransmitter systems [66–68].

Notably, the experiments with multitrode recordings under urethane anesthesia demonstrated that when the mice were injected with LPS *while they were under urethane anesthesia*, the early effects seen in LFP were completely absent both in spectral power and entropy. This striking observation can be explained by two previously discovered modes of action of urethane: (1) it potently activates the central cholinergic nuclei in the basal forebrain which innervate both HC and NCX [40], and (2) is known to protect animals from otherwise lethal LPS endotoxemia together with reducing TNFα [41]. What makes the anesthetic action of urethane unique, and important in the present work, is that it produces sleep-like cyclicity in cortical and hippocampal networks via ascending cholinergic tracts [40, 69, 70]. As is evident, this mode of action is fully compatible with our key proposal that the absence of LPS effects in urethanized animals is indeed most likely due to urethane’s action on the cholinergic pathways.

Our results demonstrating sustained abnormality in hippocampal (dorsal CA1) activity and suppression of SPW-R by LPS-induced neuroinflammation and sickness behavior at 24 hpi have important implications. First, this lends further support for the identification of the cholinergic system as the key mediator of the effects of LPS on cortical activity, because suppression of SPW-R is a direct effect of upstream cholinergic activation [62]. SPW-R in the dorsal CA1 have an established role in consolidation of memories in both rodents and men [42, 71, 72], and they participate in regulation of metabolic functions as well, such as peripheral glucose allostasis [73].

Surprisingly, LPS increases slow-wave activity in DG, contrary to the strong attenuation seen in CA1 and NCX at this timepoint both in freely moving and anesthetized animals. Furthermore, we show that animals surviving a single bout of systemic inflammation exhibit decreased threshold to kainate seizures, indicating protracted hyperexcitability especially in the DG. Considering the cholinergic mechanism postulated above, it is intriguing that systemic administration of cholinergic agonists such as pilocarpine are well-known to induce seizure activity [74]. The present data also demonstrate that the LPS-induced hypothermia has a strong seizure-suppressing effect, as seen in the generation of seizures and even lethal SE upon gradual rewarming of the animals towards normothermia.

Apart from the novel data on the earliest cytokine responses (20 min after LPS) discussed above, our results on subsequent cytokine dynamics were similar to those published before in several studies (see e.g., [19, 20]). In keeping with the mRNA copy numbers from hippocampal tissue by 2 h after LPS in Duan et al. [20], we find elevated CCL2 and IL-6 protein concentration in hippocampus (our data collected already at 1 hpi), but in contrast to their work, our results also show an increase in hippocampal TNFα both at 1 and 3 hpi. Skelly and colleagues [19] instead find increased TNFα, IL-6 and IL-1β mRNA transcription in both blood plasma and hippocampal tissue by 2 hpi, which is completely in line with our protein concentration results.

From a translational perspective, the present results are relevant for a wide variety of conditions involving innate immune response in the CNS, such as sepsis, viral infections, traumatic brain injury, stroke and epileptic seizures. Interestingly, patients with severe COVID had a cytokine profile highly similar to our LPS-injected mice at 1 hpi, when mode IV was already fully developed: robustly increased IL-6, TNFα and CCL2 plasma concentrations with IL-1β at control level [75]. Conditions leading to a pronounced activation of innate immunity increase the risk of subsequently developing chronic cognitive disability, e.g., Alzheimer’s disease [76–78], and neuroinflammation is a common pathological hallmark in clinical neurodegeneration [79]. As a whole, our data suggest that systemic-to-brain inflammatory signaling, probably mediated by cholinergic drive, potently affects the function of HC and NCX by ways which promote the acute cognitive dysfunction, delirium and ‘brain fog’ often described by severely ill patients [11, 80]. An intriguing question for future work is how the earliest and robust cortical responses identified in the present work might modify top-down control of diverse immune processes [25].

## Methods

### Animals and lipopolysaccharide injections

All animal procedures were executed in accordance with institutional guidelines and approved by the local authorities (Approval number ESAVI/7008/2020). Adult (3–7 months) C57BL/6J male and female mice from Charles River Laboratories (Sulzfeld, Germany) were used. Animals were housed under a 12-h light/dark cycle (lights on 6:00–18:00), and provided with food and water ad libitum. After surgical procedures or lipopolysaccharide injections, mice were individually housed.

To induce a systemic inflammation, mice were injected i.p. with Gram-negative bacterial endotoxin (lipopolysaccharides (LPS), serotype Salmonella enterica abortus equi, Sigma Aldrich, product number L5886). Injected dose was 0.5–2 mg/kg of LPS in saline (total injection volume 150 µl). Control animals were injected with the same total volume of nonpyrogenic saline. Injected animals were monitored with regular measurements of body temperature and weight. Dehydration and starvation of the sick animals was prevented by placing hydrogel and food pellets at the cage bottom in immediate proximity to the animals to ensure easy access.

### Chronic hippocampal LFP recordings from freely moving animals

Prior to surgery, custom-made electrodes were prepared from 50 µm tungsten wire with PTFE insulation (Advent, catalogue no. W558411). Three such wires were glued together with self-adhesive resin cement (RelyX Unicem 2 Automix) at tip intervals of 500 µm to create a linear 3-electrode probe and connected to a Millmax connector. The ground and reference electrodes were prepared from steel screws (diameter 800 µm). Electrolysis of water and impedance measurements were used to test each recording contact for free conductivity and absence of short circuits between contacts (only electrodes with contact impedance below 100 kΩ were accepted to use).

Surgical anesthesia was induced with 4% isoflurane/air mixture and maintained with 1.8–2.0% of the same mixture. The head of the animal was fixed into the stereotactic frame with earbars and biting plate. The scalp was locally anesthetized with 5 mg/ml bupivacaine hydrochloride and carefully incised at the midline and detached from the skull without damaging neck musculature. Two openings were carefully drilled in the skull without damaging the dura at (1) AP − 2.0 mm, ML 1.5 mm for hippocampal electrodes and (2) AP + 2.0, ML 1.5 mm for an epidural screw. Openings were also drilled above cerebellum for the reference and ground electrodes. The custom-made hippocampal probe was connected to a recording system of Intantech RMD2000 pre-amplifier and OpenEphys acquisition board, which were used to intraoperatively record continuous LFP and guide the probe insertion to the target position. The probe was dipped into Neurotracer DiI for later histological confirmation of its position, and subsequently slowly descended into hippocampus with a Narishige micromanipulator targeting the CA1 pyramidal layer, which was considered hit when the middle of the three electrodes showed a signal with high-frequency unit activity at a depth of 1200–1300 µm. The probe was then fixed in place with resin cement hardened by ultraviolet light. Carprofen analgesia (5 mg/kg subcutaneously) was administered at the start of operation and once per day for the first two postoperative days. The hippocampal electrodes were implanted into 15 adult mice, and a subset of them (n = 8) were also implanted with the epidural screw for recording frontal electrocorticogram (ECoG).

The experimental recordings were performed in a 25 cm × 25 cm plastic open cage with bedding cover on the floor. All recordings were videotaped and the video signal was time-synchronized with the LFP signal in Spike2 software v9.08 (Cambridge Electronic Design) using a TTL-directed LED in the videorecorded field. LFP and ECoG signals were recorded using a field potential amplifier (npi electronic EXT-16DX [bandwidth 1–500 Hz]). Signals were digitized at 8 kHz with a Cambridge Electronic Design Micro1401-3 converter. Most recordings (13/15 animals) were referenced to the cerebellar reference electrode while the rest were referenced to the ground. The cage bedding was changed after each recording day and food pellets were randomly placed into the bedding so that the animals would have a tendency to explore the cage at the start of each recording day. Each animal first had a ‘naïve baseline’ recording without preceding injection. Most of the animals (11/15) were then injected with saline on a following day, starting the recording immediately after the injection. LPS was injected on a subsequent day, after which the brain activity was recorded for approximately 3 h immediately after the injection and at 24 h intervals for the next 2 or 3 days for longitudinal follow-up.

### Automated motion tracking and quantification of traveled distance

We used DeepLabCut, a deep neural network performing markerless pose estimation [81], to track the motion of the recorded mice and enable quantification of their traveled distance over the recording period. We compressed our video recordings to 256 × 256 pixel size, and used in total 120 labeled frame images sampled randomly from six recording videos to train the network to detect the position of the mouse. After training the network was able to detect the target position (center of the back of the animal) at a mean average Euclidean error of 3.09 pixels in the test set. The trained neural network then analyzed all videos from both experimental conditions with no human intervention. DeepLabCut provides a likelihood metric for the probability of correct positional estimation, which was ~ 1 (full confidence) in > 99% of the recording time. We also used DeepLabCut to label the analyzed videos with its positional estimator as a dot, and three independent observers confirmed visually that the estimations were accurate (Additional file 3: Video S1). We approximated the traveled distance as a cumulative sum of Euclidean distances calculated from the change of x and y coordinates in subsequent time points.

### Acute hippocampal LFP measurements under urethane anesthesia

Anesthesia was induced with 4% isoflurane/air mixture followed by 1.3 g/kg of urethane injected i.p. to produce a long-lasting unconscious state with sleep-like brain state alterations [69, 82]. The depth of anesthesia was controlled by testing the absence of peripheral reflexes of the animal, and a 0.3 g/kg bolus of urethane was given if animal showed signs of elevating level of consciousness, such as muscle movements or vocalizations. Surgery was identical to the chronic implantations except that the opening for the cortical screw was omitted, and the ground and reference electrodes were prepared from TEFLON-insulated 125 µm silver wire. Hippocampal LFP was recorded using a linear 16-channel silicon probe (Neuronexus A1 × 16-5 mm-100–177), which was slowly descended into the dorsal hippocampus to maximal (tip) depth (DV) of 2.1–2.2 mm corresponding to the DG hilus. Correct position was confirmed from the characteristic neurophysiological signals from hippocampal subfields and known anatomical distances: dentate spikes identifying the DG hilus, and SPW-ripples identifying CA1. For some animals, the probe was also dipped into Neurotracer DiI before insertion and the probe position was later histologically confirmed. LFP signals were amplified with Neuralynx HS-18 amplifier and digitized with Neuralynx Digital Lynx SX acquisition system at 30 kHz.

### Behavioral measurements

Animals arrived to the behavioral facility for one week prior to the start of any experimental procedures, and were housed in groups of 4–5 during this time. We then separated the animals to individual home cages, in which they were habituated for one additional week. Measurements of baseline food and water consumption were done during the habituation week.

Motivational behavior (food and water consumption, and nest building): baseline food and water consumption were measured once daily for 1 week prior to the injections to quantify baseline level for each individual animal, and then subsequently for 1 week after the injection. Values were normalized to the mean daily consumption of the saline group over the habituation period prior to injections. Nest building was assessed as described in [83] applied to the context of this study by using Nestlet (Ancare, Bellmore, NY); briefly, baseline nest building tendency was measured during 2 days prior to injection, after which a new assessment was made at 24, 48 and 72 h after the injection. Nest quality was normalized individually to the nest produced on the second baseline day (the day immediately prior to injection). Mice which were not able to build a baseline nest of higher score than 2 were discarded from results.

Body weight: animals were weighed immediately prior to injection, 24 h after the injection and subsequently at 24 h intervals until 7 days after the injection. Rectal temperature: baseline temperature was measured with BAT-12 probe (Physitemp Instruments Inc.) at a standardized depth from the rectum immediately prior to injection, and 24 and 48 h after the injection. These measurements were not pursued further than 48 h as all animals had recovered to the baseline temperature by that time point (except for one outlier as shown in Fig. 4).

### Quantification of blood and brain cytokines

Mice were rapidly anesthetized with halothane and decapitated. Blood was sampled directly from the trunk into tubes coated with EDTA to prevent coagulation and cOmplete protease inhibitor (25x) was added. Samples were kept on ice during the whole procedure. Blood tubes were centrifuged for 10 min at + 4 °C at 1300 G. Supernatant plasma was collected and frozen in liquid nitrogen. Hippocampus was dissected immediately after decapitation and snap-frozen in liquid nitrogen, and stored at -80 °C until the analysis. On the day of the analysis, hippocampi were homogenized in RIPA buffer containing cOmplete protease inhibitor. Cytokine concentrations were determined using Cytometric Bead Array (CBA) Flex kits (BD Biosciences) for mouse MCP-1 (i.e., CCL2), IL-6, TNFα, IL-1β and IFNγ according to the manufacturer’s instructions. The samples were analyzed using BD Accuri C6 Plus flow cytometer with BD CSampler Plus software (BD Biosciences). Median PE-Height fluorescence intensity values for each bead population estimated by FlowJo software (BD Biosciences) were used to construct the standard curves. Concentration values were derived from standard curves using linear regression.

### Data analysis of LFP and ECoG signals and statistics

The LFP and ECoG signals were analyzed with custom-written scripts on MATLAB R2022b (MathWorks). First, signals were downsampled to 1000 Hz. Spectrograms were created using function mtspecgramc from Chronux toolbox (v.2.12 v03; http://chronux.org/; Observed Brain Dynamics, Partha Mitra and Hemant Bokil, Oxford University Press, New York, 2008) using 30 s windows and 3 s separation between adjacent windows. The bandwidth for analysis in the freely moving mice was 1–140 and 1–60 Hz for LFP and ECoG signals, respectively. Multitrode recordings in the urethane-anesthetized mice permitted data analysis within 1–250 Hz. Spectral entropy was calculated from the spectrograms using Matlab default function pentropy. Power spectra were calculated using Matlab default function pwelch. For the offline analysis of SPW-R, the raw signal was first filtered to the ripple frequency band (120–250 Hz) with Matlab function eegfilt (Scott Makeig, SCCN/INC/UCSD, La Jolla, 1997; https://sccn.ucsd.edu/~arno/eeglab/auto/eegfilt.html). Ripple events were detected using a power threshold of + 7 SD above mean for the absolute values of Hilbert-transformed signal of the ripple band, which was scanned in 300 ms windows with 50 ms steps (subsequent windows having a 250 ms overlap) [84]. Detected events were further required to retain ripple band power above a threshold of + 3 SD above mean for 10 ms after the crossing the detection threshold to exclude artifacts, and a minimum time span of 30 ms was required between subsequent detected ripples for classifying them as separate events. When an event passing both of these threshold criteria was detected, its peak-to-peak amplitude was determined as the difference of positive and negative peaks, and the median value of the amplitude distribution as well as the event frequency over the sampling period was calculated. Statistical tests were performed using GraphPad Prism v8.4.2 (GraphPad Software). All information on statistical tests is provided in figures and corresponding legends. All data are presented as mean ± SEM unless otherwise stated.

## Supplementary Information

**Additional file 1: Figure S1.** Spectral composition of the four cortical modes (active/mobile, SWS, REM and inflammation) and the distribution of their prevalence.** A**. Raw data taken from LFP recordings in the HC CA1 and ECoG recordings in the NCX. **B**. Prevalence distribution of the cortical modes over a 10-minute sampling period centered at 80 minutes after the start of recording (non-injected baseline) or experimental injection (saline or LPS). **Figure S2.** Neuroinflammation at 24 hpi enhances slow-wave activity in DG, while it diminishes cortical up-down states. A. (*Left*) Power spectra of DG LFP 24 hpi saline vs LPS; smaller insets displaying the difference (LPS group – saline group) in dB across the whole frequency band 1–200 Hz and zoomed to 1–10 Hz; statistical testing by unpaired t-test; n is 6 and 11 for saline and LPS, respectively. (*Right*) Representative spectrograms of DG LFP over 20 min period in saline and LPS injected mice, and 10 s insets of raw traces. B. (*Left*) Power spectra of NCX LFP 24 hpi saline vs LPS; smaller insets displaying the difference (LPS group – saline group) in dB across the whole frequency band 1–200 Hz and zoomed to 1–10 Hz; statistical testing by unpaired t-test; n is 3 and 5 for saline and LPS, respectively. (*Right*) Representative spectrograms of NCX LFP over 20 min period in saline and LPS injected mice, and 10 s insets of raw traces.

**Additional file 2. Table S1.** Statistical test values and n numbers for Fig. 4.

**Additional file 3: Video S1. **A representative sample video of a mouse exploring the experimental cage overlaid with automated tracking as shown in red and purple dots. Red dot tracked the mid-back region of the mouse and the purple dot tracked its head. Only the red dot (mid-back region) was used in quantification of the travelled distance as the position of the head was frequently masked.

## Data Availability

All data are available from the corresponding author upon reasonable request.
