# Supplementary material for: A brain cytokine-independent switch in cortical activity marks the onset of sickness behavior triggered by acute peripheral inflammation
Source: J Neuroinflammation. 2023 Jul 28;20:176. doi: 10.1186/s12974-023-02851-5 (PMC10375675; doi:10.1186/s12974-023-02851-5)
Supplement: Supplementary file 1 — Additional file 1: Figure S1. Spectral composition of the four cortical modes (active/mobile, SWS, REM and inflammation) and the distribution of their prevalence. A. Raw data taken from LFP recordings in the HC CA1 and ECoG recordings in the NCX. B. Prevalence distribution of the cortical modes over a 10-minute sampling period centered at 80 minutes after the start of recording (non-injected baseline) or experimental injection (saline or LPS). Figure S2. Neuroinflammation at 24 hpi enhances slow-wave activity in DG, while it diminishes cortical up-down states. A. (Left) Power spectra of DG LFP 24 hpi saline vs LPS; smaller insets displaying the difference (LPS group – saline group) in dB across the whole frequency band 1–200 Hz and zoomed to 1–10 Hz; statistical testing by unpaired t-test; n is 6 and 11 for saline and LPS, respectively. (Right) Representative spectrograms of DG LFP over 20 min period in saline and LPS injected mice, and 10 s insets of raw traces. B. (Left) Power spectra of NCX LFP 24 hpi saline vs LPS; smaller insets displaying the difference (LPS group – saline group) in dB across the whole frequency band 1–200 Hz and zoomed to 1–10 Hz; statistical testing by unpaired t-test; n is 3 and 5 for saline and LPS, respectively. (Right) Representative spectrograms of NCX LFP over 20 min period in saline and LPS injected mice, and 10 s insets of raw traces. [file 12974_2023_2851_MOESM1_ESM.docx]

**Additional Information**

**A brain cytokine-independent switch in cortical activity marks the onset of sickness behaviour triggered by acute peripheral inflammation**

**Samu N Kurki^1,2*^, Tommi Ala-Kurikka^1,2^, Arto Lipponen^3^, Alexey S Pospelov^1.2^, Taisia Rolova^2^, Jari Koistinaho^2^, Juha Voipio^1^, Kai Kaila^1,2^**

1. Faculty of Biological and Environmental Sciences, Molecular and Integrative Biosciences, University of Helsinki, Helsinki, Finland.

2. Neuroscience Center (HiLIFE), University of Helsinki, Helsinki, Finland.

3. Department of Psychology, University of Jyväskylä, Jyväskylä, Finland

*****Correspondence: Samu N. Kurki, MD, Faculty of Biological and Environmental Sciences, Molecular and Integrative Biosciences, and Neuroscience Center (HiLIFE)

PO Box 64, 00014 University of Helsinki, Finland, email: [samu.q.kurki@helsinki.fi](mailto:samu.q.kurki@helsinki.fi)

**Figures**


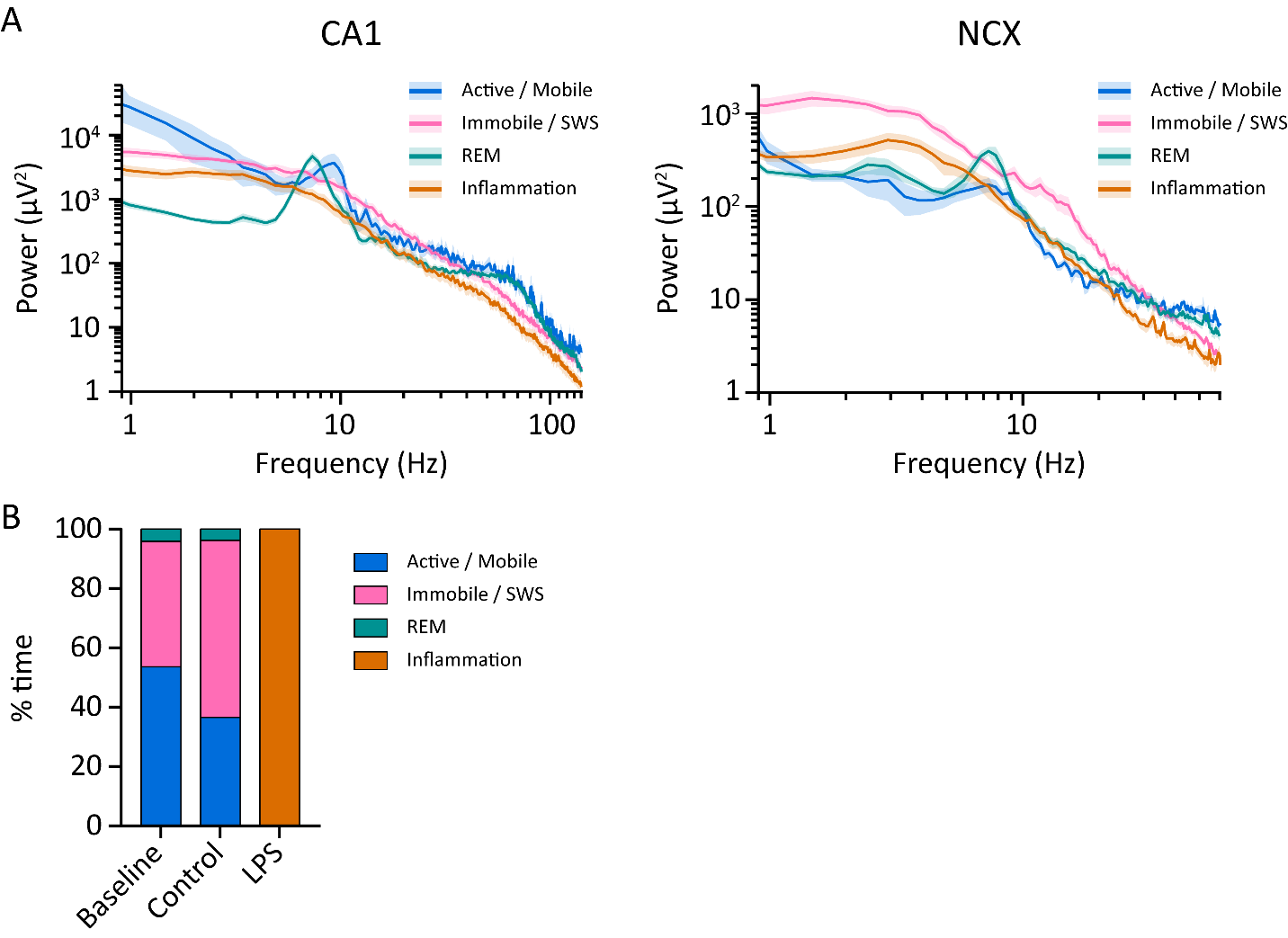


**Figure S1: Spectral composition of the four cortical modes (active/mobile, SWS, REM and inflammation) and the distribution of their prevalence.**

**A**. Raw data taken from LFP recordings in the HC CA1 and ECoG recordings in the NCX. **B**. Prevalence distribution of the cortical modes over a 10-minute sampling period centered at 80 minutes after the start of recording (non-injected baseline) or experimental injection (saline or LPS).


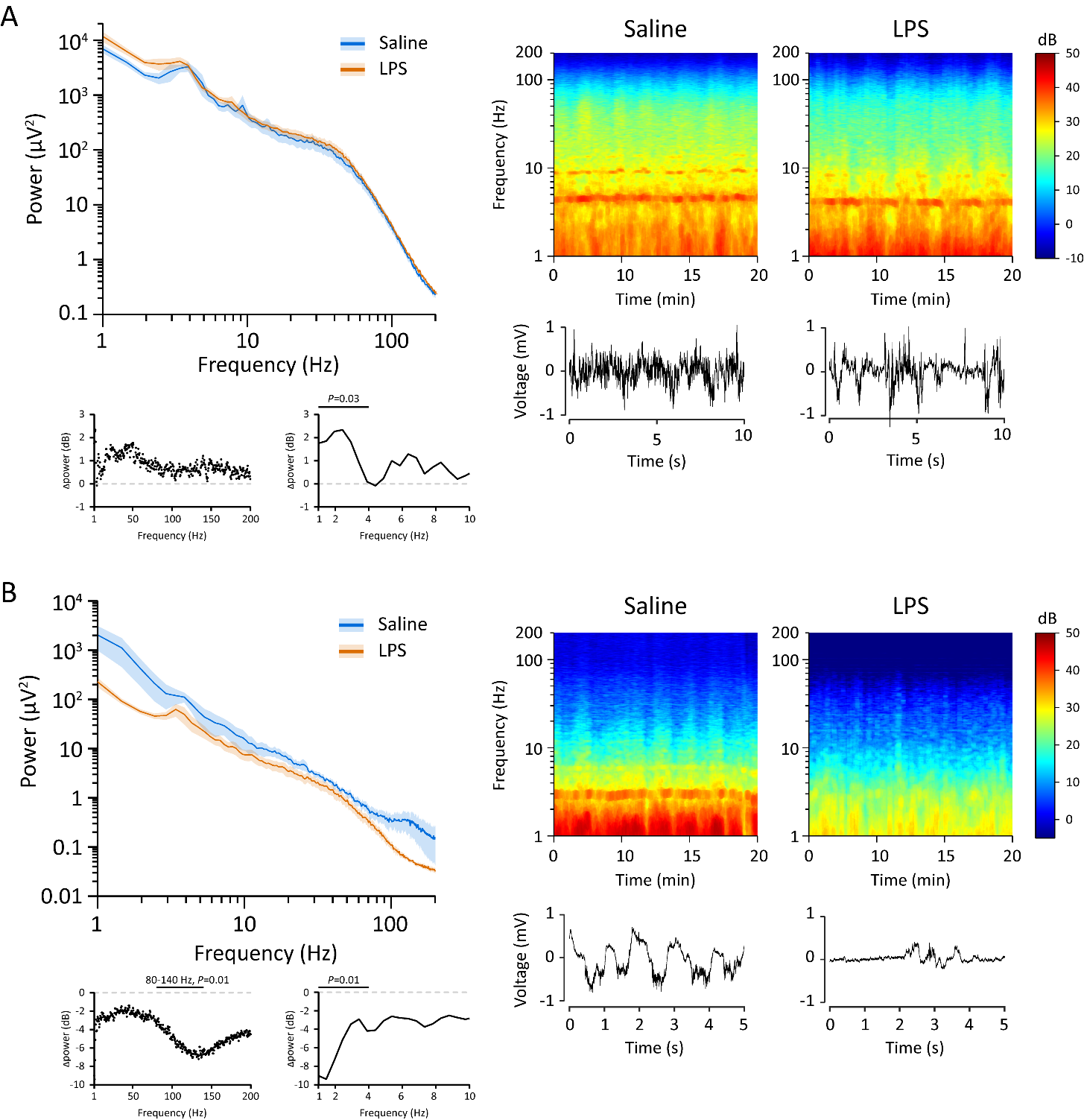


**Figure S2: Neuroinflammation at 24 hpi enhances slow-wave activity in DG, while it diminishes cortical up-down states**

A. (*Left*) Power spectra of DG LFP 24 hpi saline vs LPS; smaller insets displaying the difference (LPS group – saline group) in dB across the whole frequency band 1-200 Hz and zoomed to 1-10 Hz; statistical testing by unpaired t-test; n is 6 and 11 for saline and LPS, respectively. (*Right*) Representative spectrograms of DG LFP over 20 min period in saline and LPS injected mice, and 10 s insets of raw traces.

B. (*Left*) Power spectra of NCX LFP 24 hpi saline vs LPS; smaller insets displaying the difference (LPS group – saline group) in dB across the whole frequency band 1-200 Hz and zoomed to 1-10 Hz; statistical testing by unpaired t-test; n is 3 and 5 for saline and LPS, respectively. (*Right*) Representative spectrograms of NCX LFP over 20 min period in saline and LPS injected mice, and 10 s insets of raw traces.
